# Fluorescence quenching aptitude of carbazole for the detection of nitro-aromatics: a comprehensive experimental analysis and computational studies validation

**DOI:** 10.1039/d5ra01611h

**Published:** 2025-08-20

**Authors:** Ruby Ahmed, Farman Ali, Mohammad Jane Alam, Mohd Tameem, Abid Ali, Musheer Ahmad, Mohd Adeel Khan

**Affiliations:** a Department of Applied Chemistry, Zakir Hussain College of Engineering and Technology, AMU Aligarh 202002 India farmanfrequent@gmail.com mjalamamu@gmail.com ahmedruby1974@gmail.com abidaliamu4@gmail.com tameemkhan04@gmail.com amusheer4@gmail.com khan.adeel15@gmail.com

## Abstract

Fluorescence quenching studies of carbazole with nitroaromatics were studied and compared with 2-phenyl-1*H*-phenanthro[9,10-*d*]imidazole (M1). Carbazole (CBz) can sense all nitroaromatics, in particular picric acid (PA), whereas M1 responds to the ones having acidic hydrogen. The complex formation constant (*K*_S_) and limit of detection (LOD) were measured using the Stern–Volmer plot. Time-resolved fluorescence measurements reveal that quenching is static. We show that the mechanism of quenching involves ground state complex formation. The CBz-PA complex formation involves hydrogen bonding interaction between the N–H group of CBz and nitro group of PA, whereas M1-PA involves proton transfer from hydroxyl group of PA to nitrogen of imidazole in M1.^1^ The crystal structure of the ground-state complex (CBz-PA), its topology, and Hirshfeld surface analysis are also presented. Combined experimental and quantum chemical studies and wave function analysis were performed to study the non-covalent interactions and their role in charge transfer (CT) in the CBz-PA complex.

## Abstract 2

Fluorescence quenching studies of carbazole with nitroaromatics were studied and compared with 2-phenyl-1*H*-phenanthro[9,10-*d*]imidazole (M1).

## Graphical abstract

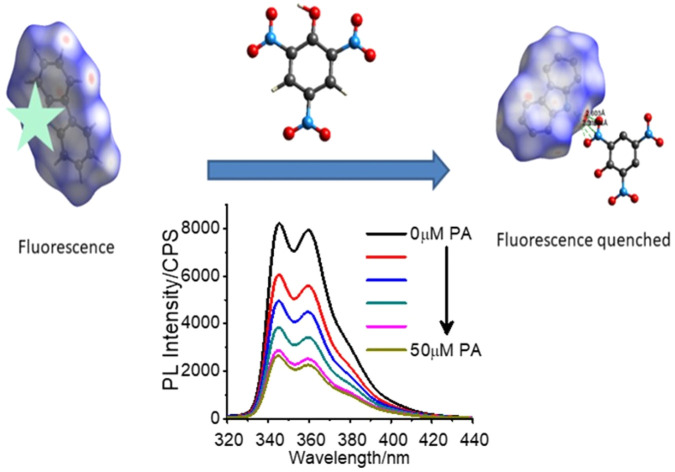

## 1 Introduction

The anthropogenic impact on the environment is a global concern of the present century. The air, water, and soil around us are continuously getting polluted with increasing chemicals.^2^ The excessive use of chemicals is affecting almost everything around us. Nitroaromatics are chemicals extensively used in dye, leather, explosives, pesticide, and pharmaceutical industries.^3^ Their over-exposure is a big nuisance to health, with symptoms like respiratory problems, eye irritation, and cancer. They are soluble in water owing to the presence of nitro groups. These compounds are thermally unstable, which is why they have sometimes been misused in explosive devices. The high reactivity, explosive nature, and low degradation rate give them a place in the list of hazardous chemicals. Hence, quantitative detection of nitroaromatics in the environment is one of the major concerns for scientists/researchers in environmental sciences.

Several methods for detecting explosives and nitroaromatic compounds include trained canines,^4^ metal detectors, imaging, and electrochemical techniques.^2,5,6^ These methods are not very attractive as their operation is tedious and trained experts must do the job.^7,8^ Fluorescence technique is an important alternative to overcome these difficulties. Fluorescence spectrometer uses photomultiplier tubes which can detect very low optical signals. It provides high sensitivity and also fast signal processing.^9–11^ Apart from imparting high sensitivity, fluorescence-based chemo sensing is also selective and rapid.

According to the literature, various fluorophores used are small molecules, supramolecules, conjugated polymers, aggregation-induced emission (AIE) active fluorescent materials, *etc*^12^. Several polyaromatic compounds, such as anthracene and pyrene derivatives have proved as excellent fluorophores for sensing nitroaromatic compounds.^13^ Nitrogen-containing heterocycles have also been explored for the sensing of picric acid. These include phenanthroimidazoles, polycarbazoles, fluorescent conjugated polymers,^14^ MOFs,^15,16^ nanomaterials, *etc.* In all the nitrogen-containing heterocycles, the fluorescence quenching was selective to PA. Fluorescence quenching of nitroaromatics other than PA or the ones without acidic hydrogen was not observed.^17–19^ However, they are equally important. Also, no rational strategy for using fluorescence quenching is presented to distinguish PA from compounds like nitrobenzene. Herein, we use carbazole and M1 for sensing of nitroaromatics. To the best of our knowledge, the fluorescence quenching in carbazole with nitroaromatics has not been reported in detail. Also, in literature, the mechanism of fluorescence quenching in nitrogen-containing heterocycles is mainly ascribed to energy transfer, proton transfer, *etc*^20^. In our recent work, we have demonstrated proton transfer leading to fluorescence quenching in M1. Herein, we made a comparative study of the sensing of nitroaromatic compounds using carbazole and M1. We also compared the mechanism of sensing in the two heterocycles. Using these two fluorophores, all nitroaromatics can be sensed and PA can be distinguished from other nitroaromatic compounds.

## 2 Experimental section

### 2.1 Chemicals and instruments

Carbazole (Merck), picric acid (Merck), and ethanol (Merck) were used as received. PerkinElmer IR spectrometer was used to record Fourier-transform infrared spectra. UV-Visible spectral measurements were carried out using a PerkinElmer LAMBDA-45 spectrophotometer. A HITACHI-F2500 spectrofluorometer was used to obtain the PL spectra. Melting points were measured in the Mel Temp device using sealed capillaries.

### 2.2 Computational details

All the reported DFT calculations have been performed using the Gaussian-09 software package.^21^ Geometry optimizations were performed at the DFT level of theory using the dispersion corrected B3LYP and CAM-B3LYP functionals^22^ along with 6-311G* basis set, which was used for all atoms. During geometry optimization, no symmetry constraints were used. For the optimized structures, analytical frequency calculations were also performed to ensure the global minimum (no imaginary frequencies). The optimized geometry was subjected to a time-dependent density functional theory (TD-DFT) computation at the B3LYP/6-311G* level of theory to obtain UV-Visible absorption data for complex and its constituent molecules respectively. The CAM-B3LYP functional, combination of hybrid B3LYP and the long-range correction was employed specifically to calculate the frontier molecular orbitals (HOMO–LUMO) for CBz-PA complex, owing to its better treatment of charge transfer excitations. The Integral Equation Formalism Polarizable Continuum Model (IEFPCM) was also applied throughout the calculations to account for the solvent effects. It was also used to perform Natural Transition Orbital (NTO) analysis, providing clear visualization of electron–hole pairs and helping to understand charge transfer behavior in the electronic transitions.

### 2.3 General procedure of synthesis of the compound

**Scheme 1:**
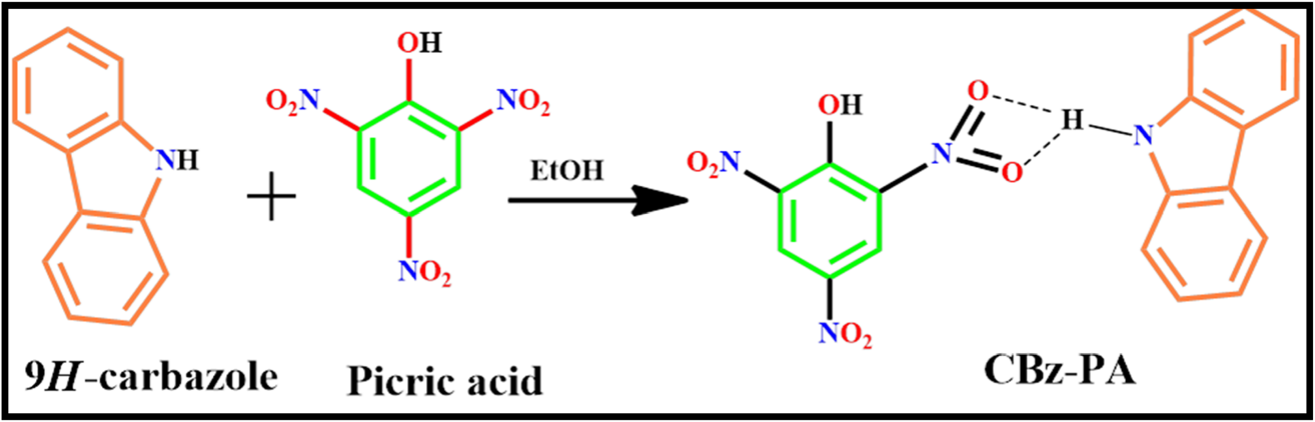
Synthesis of CBz-PA.

Crystals of CBz-PA were synthesized by dissolving carbazole and picric acid in ethanol. 20 mg of carbazole (0.1197 mole), 20 mg of picric acid (0.087 mole) were dissolved in 5 mL of ethanol. The solution was left undisturbed. Fine needle-shaped, brown crystals were obtained in good yield. Crystals of M1-PA were also obtained similarly (Scheme 1).^1^

## 3 Results and discussion

### 3.1 Crystal structure description

**Fig. 1:**
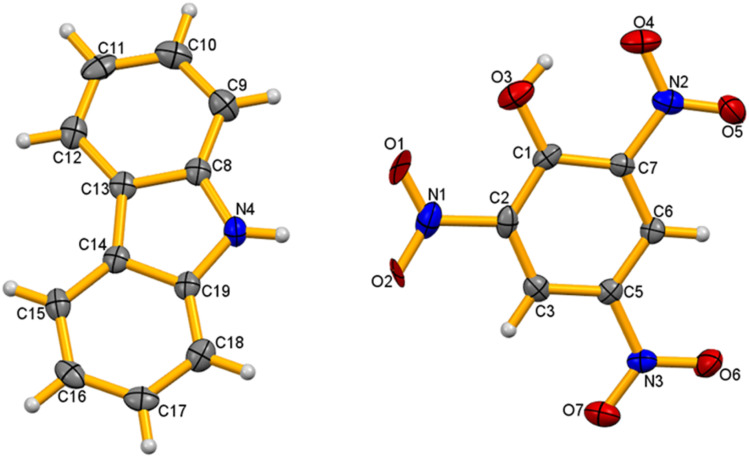
Ortep view of the co-crystal CBz-PA.

**Fig. 2:**
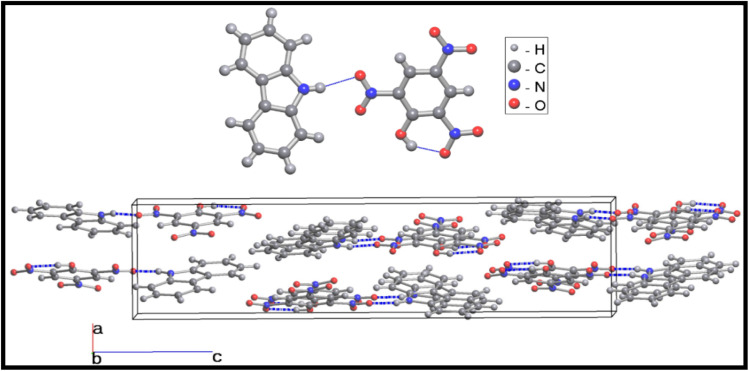
The hydrogen-bonded dimer of carbazole and picric acid (top) and the packing of the dimers in the unit cell (bottom). The hydrogen bonds are highlighted in blue.

The single-crystal X-ray structure determination shows that the co-crystal CBz-PA crystallizes in an orthorhombic system, with space group *P*2_1_2_1_2_1_. The asymmetric unit of co-crystal consists of two different organic moieties (Fig. 1), *i.e.*, picric acid and carbazole. The crystal packing structure (Fig. 2) is stabilized *via* intricate hydrogen bonding array and strong π⋯π interactions. The –NO_2_ group of picric acid is connected to the amine hydrogen of ligand L1 *via* H-bonding interactions N4–H4⋯O1 = 2.53 Å and N4–H4⋯O2 = 2.56 Å and C12–H12⋯O5 = 2.584 Å, resulting in a zig-zag architecture. Multi-point π–π interactions give rise to a layered supramolecular structure.

### 3.2 Hirshfeld surface analysis

**Fig. 3:**
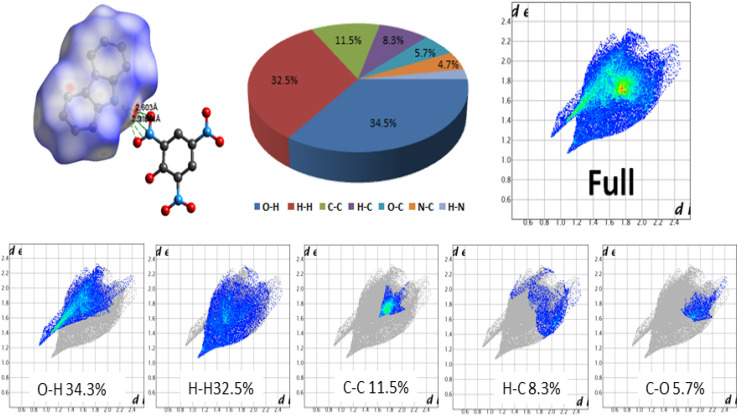
In a *d*_norm_ surface, red spots show strong hydrogen bonds between acceptor and donor atoms, white light red spots indicate weak intermolecular interactions. The *d*_norm_ plots were plotted with a color scale between blue and red from −0.376 to 1.134.

Hirshfeld's surface provides an extended qualitative and quantitative analysis of the interactions between the constituents of the complex.^23,24^ The analysis of various interactions in CBz-PA reveal the presence of N4–H4⋯O1, N4–H4⋯O2, N4–H4⋯N1, hydrogen bonds, leading to the formation of CBz-PA dimer, a three-dimensional complex. Red spots in the Hirshfeld surface of the carbazole unit are centered at N4H4 (Fig. 3).

This red spot has a picric acid molecule in its vicinity, which forms electrostatic interaction between carbazole unit and picric acid molecule. The percentage contribution of various interactions is shown in the fingerprint (FP) plot. O⋯H and H contacts percentage contributions dominate, 34.3% and 32.5%, respectively. As is revealed by the structure, there are intermolecular as well as intramolecular hydrogen bonds. Intramolecular hydrogen bonds are found in the picric acid unit, between O1O3 and O4O3 atoms, of bond length 2.541 Å and 2.547 Å, respectively.

### 3.3 Photophysical characterization

**Fig. 4:**
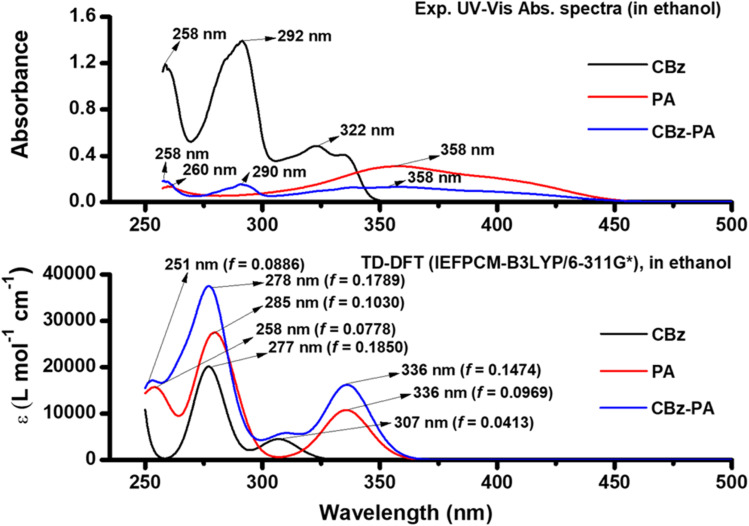
Comparison of UV-Vis absorption spectra of CBz-PA, recorded in EtOH (1 × 10^−4^ M), with the spectra (*ε*-molar absorptivity, *f*-oscillator strength) obtained from TD-DFT calculations in solution phase.

**Fig. 5:**
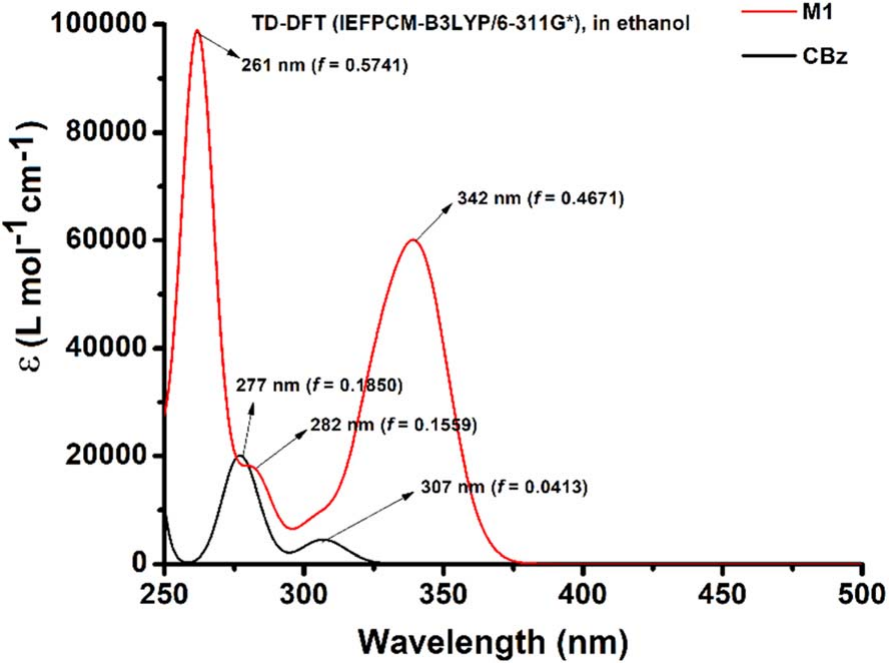
Theoretical UV-Vis absorption spectra for M1 and CBz.

**Fig. 6:**
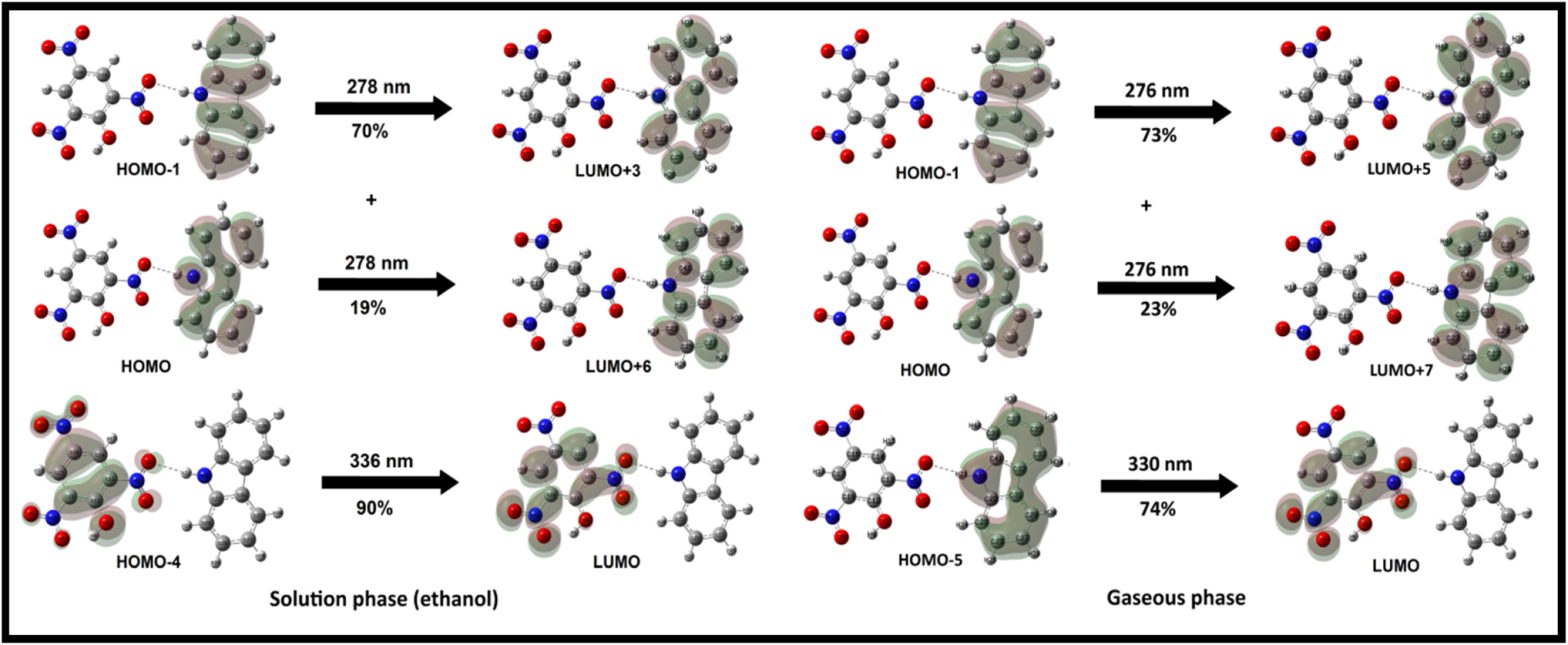
Molecular orbitals involved in electronic transitions (with major contributions >10%) for UV-Vis spectrum of CBz-PA in solution phase.

TD-DFT calculations were used to characterize the UV-Visible absorption bands. A complete set of TD-DFT calculations were performed on M1, CBz, PA, and CBz-PA, at the TD-IEFPCM-B3LYP/6-311G* level of theory in solution phase (ethanol) to characterize their singlet excited states and electronic properties. The comparison of experimental UV-Visible absorption spectra of carbazole, picric acid, and CBz-PA recorded in ethanol to those obtained by TD-DFT calculations in solution phase is shown in Fig. 4. Also, the comparison of UV-Visible spectrum of M1 with that of CBz is presented in Fig. 5. The assignment of the observed bands has been done in terms of molecular orbitals (MOs) by matching the maximum absorption peak with the oscillator strength values. The absorption spectrum of carbazole in ethanol exhibited two prominent absorption bands, at 292 nm and 322 nm, respectively. These bands predicted at 277 nm and 307 nm in solution phase are due to the transitions H−1 → LUMO (78%) + HOMO → L+1 (18%) and HOMO → LUMO (93%) respectively. For the case of M1, the UV-Vis bands obtained at 282 nm and 342 nm in solution phase are attributed to H−1 → LUMO (64%) + H−1 → L+1 (20%) and HOMO → LUMO (75%) + HOMO → L+1 (19%) respectively. The absorption peak for picric acid is observed at 358 nm. The corresponding band calculated at 336 nm in solution phase is assigned to HOMO → LUMO (86%). The complex CBz-PA has multiple absorption peaks. The maximum absorption peaks observed at 290 nm and 358 nm are due to carbazole and picric acid moieties. These characteristic bands obtained theoretically at 278 nm and 336 nm in solution phase are due to H−1 → L+3 (70%) + HOMO → L+6 (19%) and H−4 → LUMO (90%) respectively. However, these bands calculated at 276 nm (*f* = 0.1018) and 330 nm (*f* = 0.0990) in gaseous phase are assigned to H−1 → L+5 (73%) + HOMO → L+7 (23%) and H−5 → LUMO (74%) respectively. The spatial plots of MOs involved in these transitions are illustrated in Fig. 6.

The UV-Vis spectra obtained *via* TD-DFT match the UV-Vis spectra seen experimentally very well.

### 3.4 Theoretical studies of charge transfer in CBz-PA

**Fig. 7:**
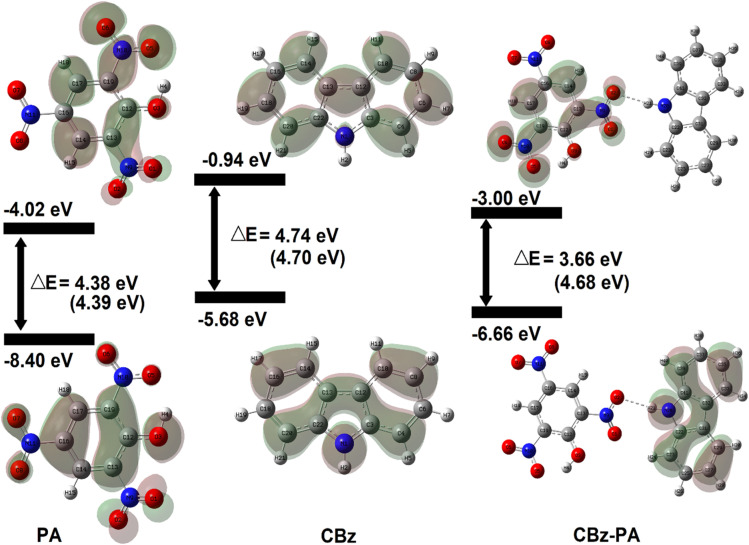
Energy diagrams and Kohn–Sham orbitals of PA, CBz, and CBz-PA (values mentioned in brackets are in solution phase).

**Fig. 8:**
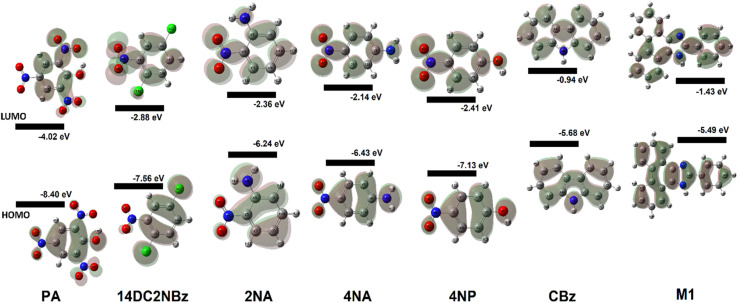
Spatial plots of HOMO and LUMO of picric acid (PA), 1,4-dichloro-2-nitro benzene (14DC2NBz), 2-nitro aniline (2NA), 4-nitro aniline (4NA), carbazole (CBz) and M1.

**Fig. 9:**
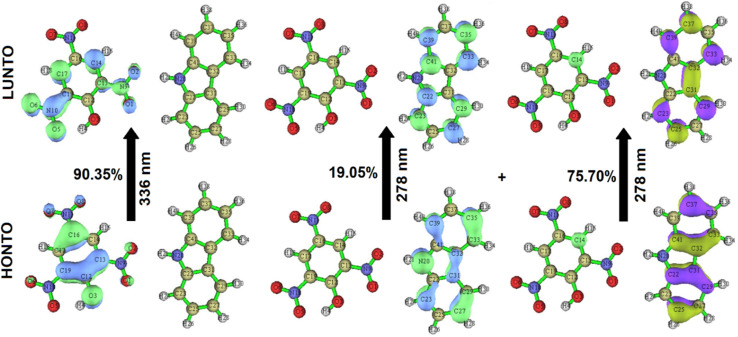
NTOs plots.

The electronic characteristics of CBz-PA were investigated using DFT calculations. The FMO analysis is performed to visualize the delocalization of MOs and charge transfer properties, Fig. 7. We know that the charge transfer process occurs from the HOMO to the LUMO as a result of the intermolecular hydrogen bonding and π–π stacking in the ground state of this donor–acceptor complex, and the electron cloud reconfigures over the donor–acceptor moieties, resulting in the formation of new molecular orbitals MOs.^25^ In the CBz-PA, HOMO and LUMO are symmetrically and spatially confined on CBz (donor) and PA (acceptor), respectively, indicating where the electron density is concentrated in these frontier MOs. Based on the distribution of HOMO–LUMO, the molecule may have good charge separation capabilities, making it a candidate for sensing application. HOMO appears to be more localized around the donor part of the molecule while LUMO extends more toward the acceptor side, indicating potential charge-transfer characteristics upon excitation. However, the HOMO to LUMO excitation was not observed for the case of CBz-PA as suggested by photophysical and MOs studies. FMOs diagrams of picric acid (PA), 1,4-dichloro-2-nitro benzene (14DC2NBz), 2-nitro aniline (2NA), 4-nitro aniline (4NA), carbazole (CBz) and M1 have also been obtained and presented in Fig. 8. NTO analysis is performed to obtain one pair of NTO (highest occupied NTO *i.e.* HONTO and lowest unoccupied NTO *i.e.* LUNTO) having eigenvalue very close to 1. HONTOs and LUNTOs of CBz-PA for states S0 → S10 and S0 → S23 were investigated. The transition between the two NTOs in this pair represents the real character of the electron excitation. The excitation at 278 nm (S0 → S23) can be regarded as transition from bonding π to antibonding π orbital of the CBz moiety, with contribution of 75.70%. From NTO eigenvalues, we also notice a transition also has small contribution (19.05%) to the excitation, Fig. 9. The excitation at 336 nm (S0 → S10) can be regarded as transition involving lone pair of O atoms to antibonding π orbital of the PA moiety, at least having contribution of 90.35%. Moreover, the Δ*r* index and Ʌ index for CBz-PA were also obtained to measure charge-transfer length (*i.e.* distance between centroids of hole and electron) and characterize electron excitation respectively. The values of Δr are found to be 0.995 Å and 1.060 Å for S10 and S23 respectively. While the Ʌ index is found to be 0.538 and 0.720 for S10 and S23 respectively. The values are suggesting the local excitation/partial charge transfer character.

### 3.5 Sensing studies and TCSPC

**Fig. 10:**
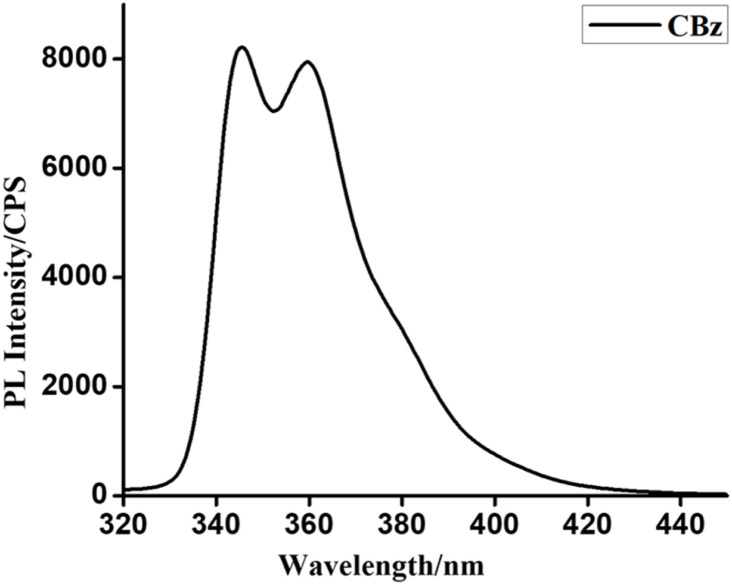
Fluorescence spectra of carbazole.

The fluorescence spectra were recorded in ethanol, at (*λ*_ex_) of 310 nm, taking *λ*_em_ in the range 350–550 nm, with a scan rate of 220 nm min^−1^ and a slit width of 3 nm. The fluorescence spectra of carbazole exhibited a peak at 359.5 nm (Fig. 10). The fluorescence quenching with various analytes was studied. The metal ions checked for fluorescence quenching include Na^+^, Cu^2+^, Mg^2+^, Fe^2+^, Cd^2+^. All the tested metal ions were unable to quench the fluorescence of carbazole. Nitroaromatic compounds tested for their quenching ability include 2-nitrotoluene, trinitrotoluene, dinitrobenzene, 1,4-dinitro-2-chlorobenzene, 2-nitroaniline, 4-nitroaniline, and 4-nitrophenol and picric acid. Benzoic acid and bisphenol were also checked. Stock solutions of the metal ions mentioned above and aromatic compounds were of 10 mM concentration. Stern–Volmer plot plotted for fluorescence intensity against quencher concentration. Stern–Volmer constant (*K*_s_) was calculated using the Stern–Volmer equation.

1 *F*_0_/*F* = 1 + *K*_S_[*Q*]

Where *F*_0_ is fluorescence intensity without quencher, and *F* is fluorescence intensity with the quencher. [*Q*] is the concentration of quencher in mole per liter.

**Fig. 11:**
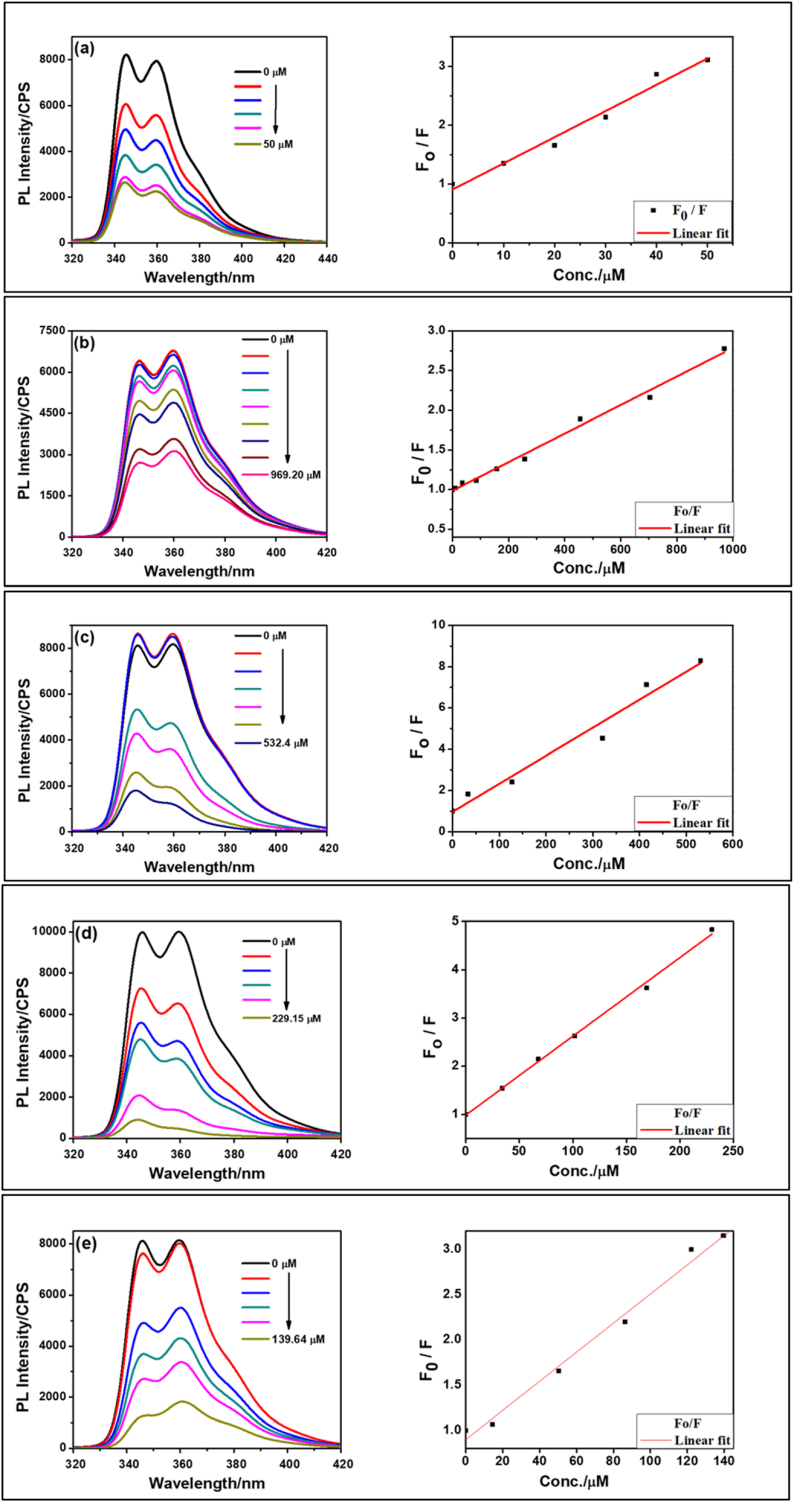
Fluorescence quenching and Stern–Volmer plots upon addition of nitroaromatic compounds to CBz. (a) Picric acid, (b) 1,4 dichloro-2-nitro benzene, (c) 2-nitro-aniline, (d) 4-nitro-aniline, (e) 4-nitrophenol.

The tested metal ions were unable to quench the fluorescence of carbazole. All the tested nitroaromatic compounds, *viz* picric acid, 1,4-dinitro-2-chlorobenzene, 2-nitroaniline, 4-nitroaniline, and 4-nitrophenol exhibited quenching of fluorescence of carbazole, with *K*_S_ values as 4.4 × 10^4^, 1.7 × 10^3^, 1.3 × 10^4^, 1.6 × 10^4^, and 1.6 × 10^4^ liter per mole, respectively. The *K*_S_ values and LOD are summarized in Table 1. Fig. 11 shows the fluorescence quenching of carbazole with nitroaromatic compounds. The *K*_S_ is highest for picric acid, having three nitro groups, and is lowest for 1,4-dinitro-2-chlorobenzene. The *K*_S_ values are similar for 2-nitroaniline, 4-nitroaniline, and 4-nitrophenol (Table 1). These values are one order less than the values reported in the literature^26^ for similar nitrogen containing heterocycles.

**Table 1:** The values of *K*_s_ and LOD

Complex	*K*_S_ (liter per mole)	LOD mole per liter, (ppm)
CBz-picric acid	4.4 × 10^4^	7.1 × 10^−6^, 1.6
CBz- 1,4-dichloro-2-nitrobenzene	1.7 × 10^3^	5.2 × 10^−5^, 10.0
CBz-2-nitroaniline	1.3 × 10^4^	1.0 × 10^−4^, 14.4
CBz-4-nitroaniline	1.6 × 10^4^	9.9 × 10^−6^, 1.3
CBz-4-nitrophenol	1.6 × 10^4^	1.2 × 10^−5^, 1.6
M1-picrate	8.6 × 10^4^	2.8 × 10^−6^, 0.6

The carbazole, however, do not respond to fluorescence quenching when benzoic acid and bisphenol were added (SI). We find that the nitro group is essential in the aromatic analyte for the fluorescence quenching of carbazole.

**Fig. 12:**
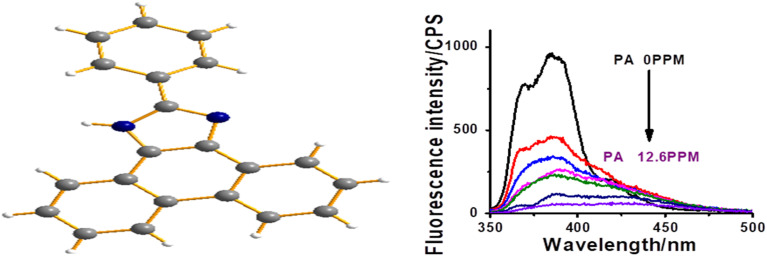
Molecular structure of M1 and its fluorescence quenching with PA.

Aromatic –NO_2_ group of the analyte enters into electrostatic interaction with the N–H bond of CBz fluorophore, resulting in the formation of a complex and thereby quenching the fluorescence of CBz. Crystal of one such complex CBz-PA is discussed above. Herein, we also wish to discuss the fluorescence quenching of M1,^1^ nitrogen-containing heterocycle, for its comparative study. The molecular structure of M1 and its fluorescence quenching with PA is shown in Fig. 12.

**Fig. 13:**
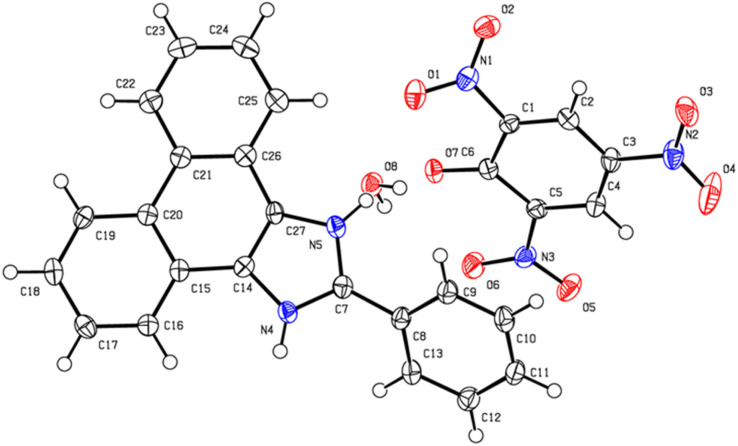
Crystal structure of M1^+^–PA^−^.

The *K*_S_ and LOD for M1 are 8.6 × 10^4^ and 0.6 ppm. These values are slightly higher than obtained in the case of carbazole. However, the fluorescence quenching in M1 is not observed with nitroaromatic compounds without acidic hydrogen. We have earlier demonstrated that acidic hydrogen is transferred from PA to nitrogen of imidazole in M1, which leads to fluorescence quenching (Fig. 13).

It may be noted that carbazole and M1 when used together for the sensing of nitroaromatics can give more effective and reliable results. This can be understood with the help of Table 2. Wherein the responses of analyte towards fluorescence quenching of CBz and M1 is recorded. Analytes only sensed by M1 but not by CBz are the ones having only acidic protons. Analytes which show fluorescence quenching of CBz as well as of M1 are the one which have both the nitro group as well as acidic proton. Analytes which respond to only CBz are nitroaromatic without acidic proton. Analytes without nitro group and acidic proton do not show fluorescence quenching of CBz and M1.

**Table 2:** Responses of analytes towards fluorescence quenching of CbZ and M1

S.no.	CBz	M1	Inference
1	No	Yes	Only acidic proton, example benzoic acid
2	Yes	Yes	Nitro group as well as acidic proton present, example PA
3	Yes	No	Only nitro group is present, example: nitrobenzenzene
4	No	No	Neither nitro nor acidic proton present, example sodium chloride

**Fig. 14:**
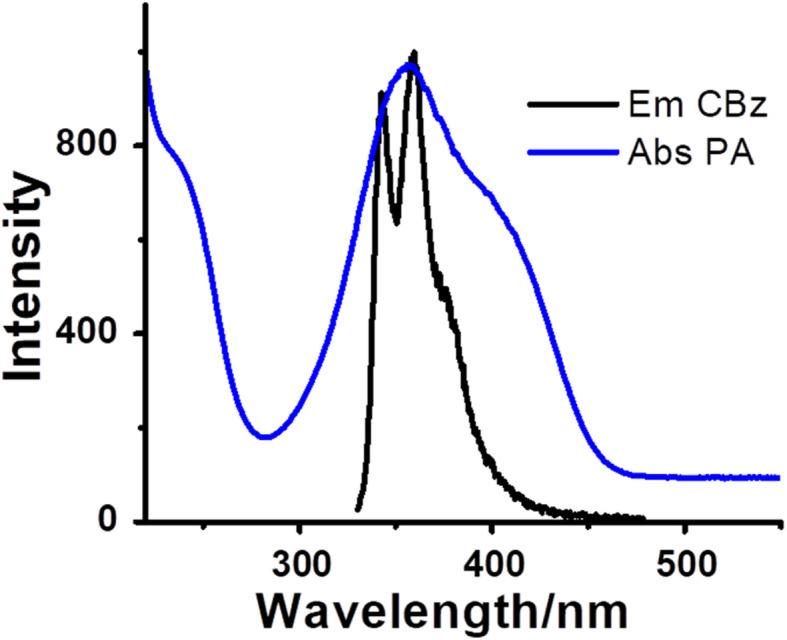
Overlap of photoluminescence spectra of CBz and absorption spectra of PA.

Fig. 14 shows the photoluminescence spectra of CBz and absorption spectra of PA. We find that the absorption spectrum of PA significantly overlaps with the emission spectrum of carbazole, illustrating that fluorescence resonance energy transfer from carbazole to PA may occur. Besides the competitive absorption and FRET mechanism, the electrostatic interactions between the sensing materials and analyte may also affect the fluorescence quenching response.

**Fig. 15:**
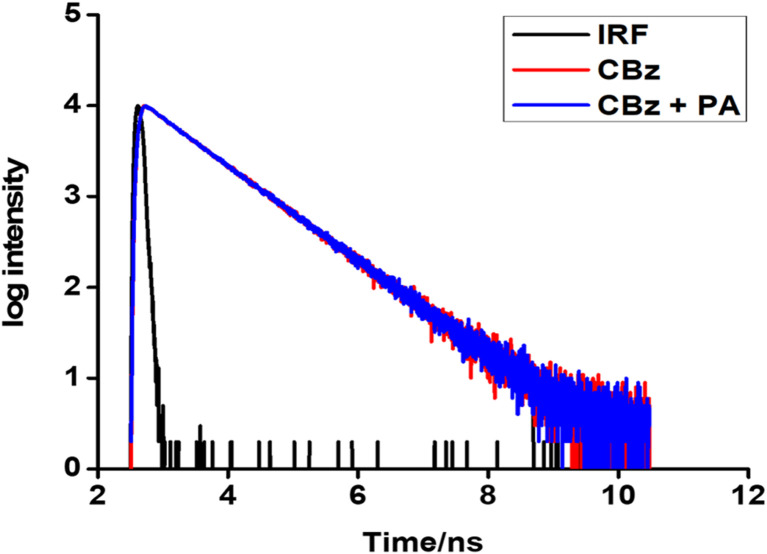
Semilog plots of fluorescence decay *versus* time for CBz-PA and CBz.

To resolve this, time-resolved photoluminescence measurements for CBz and CBz-PA were done. The average lifetime CBz and CBz-PA were 8.25 and 8.27 ns, respectively (Fig. 15). We find a little difference in their fluorescence lifetimes. This data can be used to estimate the energy transfer efficiency between carbazole and picric acid. We used the equation given below.^27^2
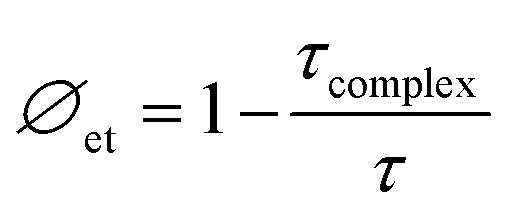


where *∅*_et_ is energy transfer efficiency, *τ*_complex_ is the average lifetime of CBz-PA, and *τ* is the average lifetime of CBz. In carbazole, *τ* is inversely proportional to the sum of the radiative (*k*_r_) and non-radiative (*k*_nr_) rate constant. For CBz-PA, *τ*_complex_ is inversely proportional to the sum of the radiative (*k*_r_), non-radiative (*k*_nr_), and energy transfer (*k*_et_) rate constants. The unchanged lifetime rules out the possibility of energy transfer involved in fluorescence quenching. In the fluorescence quenching of CBz-PA, the energy transfer efficiency was calculated as 0.23%, which is very low. The quenching involved here is static quenching. The same is also true for the fluorescence quenching of M1 with PA.

## 4 Conclusion

In summary, the fluorescence quenching of carbazole with nitroaromatic compounds has been studied and compared with M1. Carbazole can sense all nitroaromatic, whereas M1 can sense the ones having acidic hydrogen. The quenching mechanism in CBz involves electrostatic interaction between the N–H group of carbazole and nitro group of an aromatic compound resulting in the ground state complex formation. Time-resolved fluorescence measurements ruled out the possibility of energy transfer and suggested that the quenching is static. The molecule M1, however, will respond to aromatic compounds having acidic hydrogen like PA, benzoic acids and phenols, *etc.* The mechanism of quenching involved here is proton transfer from the analyte to the nitrogen atom of the imidazole in M1. This study provides a rational strategy to identify nitroaromatic compounds from other aromatic compounds and also to differentiate aromatic acids from simple nitroaromatic compounds. The high value of formation constant and low LOD reveal the potential of two fluorophores for their use in explosives detection and their differentiation. This study also enables one to design nitrogen-containing fluorophores of different colors for the sensing of nitroaromatics.

## Conflicts of interest

There are no conflicts to declare.

## Supplementary Material

RA-015-D5RA01611H-s001

RA-015-D5RA01611H-s002

## Data Availability

Crystallographic data for [Cbz-PA] has been deposited at the CCDC under [1961338]. CCDC 1961338 contains the supplementary crystallographic data for this paper.^28^ Supplementary information is available. See DOI: https://doi.org/10.1039/d5ra01611h.

## References

[cit1] Ahmed R. (2020). Phenanthroimidazole derivatives as a chemosensor for picric acid: a first realistic approach. New J. Chem..

[cit2] Wu J., Liu W., Ge J., Zhang H., Wang P. (2011). New sensing mechanisms for design of fluorescent chemosensors emerging in recent years. Chem. Soc. Rev..

[cit3] Mei J. (2014). Aggregation-induced emission: The whole is more brilliant than the parts. Adv. Mater..

[cit4] Agarwal N. (2011). Tuning of HOMO levels of carbazole derivatives: New molecules for blue OLED. Synth. Met..

[cit5] Roy B., Bar A. K., Gole B., Mukherjee P. S. (2013). Fluorescent Tris-Imidazolium Sensors for Picric Acid Explosive. *J. Org. Chem.*.

[cit6] Yinon J. (2003). Peer Reviewed: Detection of Explosives by Electronic Noses. Anal. Chem..

[cit7] Hodyss R., Beauchamp J. L. (2005). Multidimensional Detection of Nitroorganic Explosives by Gas Chromatography-Pyrolysis-Ultraviolet Detection. Anal. Chem..

[cit8] Holthoff E. L., Stratis-Cullum D. N., Hankus M. E. (2011). A nanosensor for TNT detection based on molecularly imprinted polymers and surface enhanced Raman scattering. Sensors.

[cit9] Ahamad M. N., Shahid M., Ahmad M., Sama F. (2019). Cu(II) MOFs Based on Bipyridyls: Topology, Magnetism, and Exploring Sensing Ability toward Multiple Nitroaromatic Explosives. ACS Omega.

[cit10] Ali F., Periasamy N., Patankar M. P., Narasimhan K. L. (2011). Integrated Organic Blue LED and Visible-Blind UV Photodetector. J. Phys. Chem. C.

[cit11] Ali F., Nayak P. K., Periasamy N., Agarwal N. (2017). Synthesis, photophysical, electrochemical and electroluminescence studies of red emitting phosphorescent Ir(III) heteroleptic complexes. J. Chem. Sci..

[cit12] Sun X. (2018). Preparation of cyano-substituted tetraphenylethylene derivatives and their applications in solution-processable OLEDs. Molecules.

[cit13] Li Y., Zhang W., Niu J., Chen Y. (2012). Mechanism of photogenerated reactive oxygen species and correlation with the antibacterial properties of engineered metal-oxide nanoparticles. ACS Nano.

[cit14] Shanmugaraju S., Mukherjee P. S. (2015). π-Electron rich small molecule sensors for the recognition of nitroaromatics. Chem. Commun..

[cit15] Sahoo J., Waghmode S. B., Subramanian P. S., Albrecht M. (2016). Specific Detection of Picric Acid and Nitrite in Aqueous Medium Using Flexible Eu(III)-Based Luminescent Probe. ChemistrySelect.

[cit16] Wang L. (2016). RSC Advances Star-shaped triazatruxene derivatives for rapid fl uorescence fi ber-optic detection of nitroaromatic explosive vapors. RSC Adv..

[cit17] Nath S. (2018). *et al.*, A sensitive and selective sensor for picric acid detection with a fluorescence switching response. New J. Chem..

[cit18] Kathiravan A. (2019). Pyrene-Based Chemosensor for Picric Acid - Fundamentals to Smartphone Device Design. Anal. Chem..

[cit19] Kaleeswaran D., Murugavel R. (2018). Picric acid sensing and CO 2 capture by a sterically encumbered azo-linked fluorescent triphenylbenzene based covalent organic polymer. J. Chem. Sci..

[cit20] Lakowicz J. R. , Principles of Fluorescence Spectroscopy, Third Edition, (2014)

[cit21] Golla R., Kumar P. R., Suchethan P. A., Foro S., Nagaraju G. (2020). Synthesis, photophysical, electrochemical properties and crystal structures and Hirschfeld surface analysis of 4′-dimethoxyphenyl-(2,6-di-2-pyrazinyl) pyridines. J. Mol. Struct..

[cit22] Koch U., Popelier P. L. A. (1995). Characterization of C-H-O hydrogen bonds on the basis of the charge density. J. Phys. Chem..

[cit23] Espinosa E., Alkorta I., Elguero J., Molins E. (2002). From weak to strong interactions: A comprehensive analysis of the topological and energetic properties of the electron density distribution involving X-H⋯F-Y systems. J. Chem. Phys..

[cit24] Vener M. V., Egorova A. N., Churakov A. V., Tsirelson V. G. (2012). Intermolecular hydrogen bond energies in crystals evaluated using electron density properties: DFT computations with periodic boundary conditions. J. Comput. Chem..

[cit25] Gierschner J., Park S. Y. (2013). Luminescent distyrylbenzenes: Tailoring molecular structure and crystalline morphology. J. Mater. Chem. C.

[cit26] Chowdhury A., Mukherjee P. S. (2015). Electron-rich triphenylamine-based sensors for picric acid detection. J. Org. Chem..

[cit27] Jennings T. L., Singh M. P., Strouse G. F. (2006). J. Am. Chem. Soc..

[cit28] Ahmed R. Ali F. , Alam M. J., Tameem M., Ali A., Ahmad M. and Khan M. A., CCDC 1961338: Experimental Crystal Structure Determination, 2025, 10.5517/ccdc.csd.cc23txzv

