# Supplementary material for: Fluorescence quenching aptitude of carbazole for the detection of nitro-aromatics: a comprehensive experimental analysis and computational studies validation
Source: RSC Adv. 2025 Aug 20;15(36):29479–89. doi: 10.1039/d5ra01611h (PMC12377312; doi:10.1039/d5ra01611h)
Supplement: RA-015-D5RA01611H-s001 [file RA-015-D5RA01611H-s001.pdf]

Supplementary Information (SI) for RSC Advances.
This journal is © The Royal Society of Chemistry 2025

SUPPORTING INFORMATION

3.1 Crystal structure description

The single-crystal X-ray structure determination shows that the co-crystal CBz-PA crystallizes in an orthorhombic system, with space group $P2_12_12_1$. The asymmetric unit of co-crystal consists of two different organic moieties (Figure 1), i.e., picric acid and carbazole. The crystal packing structure (Figure 2) is stabilized via intricate hydrogen bonding array and strong π…π interactions. The $–NO_2$ group of picric acid is connected to the amine hydrogen of ligand L1 via H-bonding interactions N4-H4…O1 = 2.53 Å and N4-H4…O2 = 2.56 Å and C12-H12…O5 = 2.584 Å, resulting in a zig-zag architecture (Figure 2b). Multi-point π-π interactions give rise to a layered supramolecular structure.

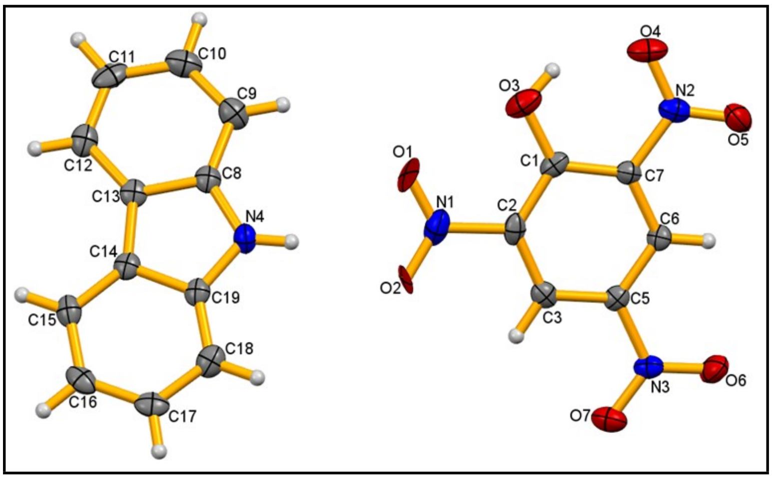

Figure 1: Ortep view of the co-crystal CBz-PA

The crystal and refinement data are collected in Table 1 for CBz-PA. The selected bond distances and angles for ligands are given in Table 2 and Table 3.

Table 1: Crystal data and structure refinement for CBz-PA

**Parameters**	
**Identification code**	**Fac97_0m_a**
Empirical formula	$C_{18}H_{12}N_4O_7$
Formula weight	396.32
Temperature/K	100(2)
Crystal system	orthorhombic
Space group	$P2_12_12_1$
a/Å	6.8385(2)

**Parameters**	
**Identification code**	**Fac97_0m_a**
b/Å	8.6736(3)
c/Å	27.7797(10)
α/°	90
β/°	90
γ/°	90
Volume/Å^3	1647.74(10)
Z	4
ρ_{calc}g/cm^3	1.5975
μ/mm^{-1}	0.126
F(000)	816.5
Crystal size/mm^3	0.39 × 0.25 × 0.19
Radiation	Mo Kα (λ = 0.71073)
2Θ range for data collection/°	5.54 to 50.1
Index ranges	-9 ≤ h ≤ 9, -11 ≤ k ≤ 11, -37 ≤ l ≤ 37
Reflections collected	26508
Independent reflections	2901 [R_{int} = 0.0438, R_{sigma} = 0.0292]
Data/restraints/parameters	2901/0/258
Goodness-of-fit on F^2	1.065
Final R indexes [I>=2σ (I)]	R_1 = 0.0647, wR_2 = 0.1667
Final R indexes [all data]	R_1 = 0.0691, wR_2 = 0.1728
Largest diff. peak/hole / e Å^{-3}	0.93/-1.00
Flack parameter	-2.6(4)

Table 2: Bond Lengths for CBz-PA

**Atom**	**Atom**	**Length/Å**	**Atom**	**Atom**	**Length/Å**
O1	N1	1.144(5)	C5	C6	1.382(5)
O2	N1	1.141(5)	C6	C7	1.375(5)
O3	C1	1.323(4)	C8	C9	1.402(5)
O4	N2	1.246(4)	C8	C13	1.416(5)
O5	N2	1.205(4)	C9	C10	1.376(6)
O6	N3	1.233(4)	C10	C11	1.384(6)
O7	N3	1.226(4)	C11	C12	1.391(5)

N1	C2	1.458(4)	C12	C13	1.396(5)
N2	C7	1.464(4)	C13	C14	1.445(5)
N3	C5	1.460(4)	C14	C15	1.399(5)
N4	C8	1.377(5)	C14	C19	1.412(5)
N4	C19	1.384(5)	C15	C16	1.384(5)
C1	C2	1.410(5)	C16	C17	1.397(5)
C1	C7	1.410(5)	C17	C18	1.382(5)
C2	C3	1.386(5)	C18	C19	1.394(5)
C3	C5	1.380(5)			

Table 3: Bond Angles for CBz-PA

**Atom**	**Atom**	**Atom**	**Angle/°**	**Atom**	**Atom**	**Atom**	**Angle/°**
O2	N1	O1	117.8(4)	C6	C7	N2	116.9(3)
C2	N1	O1	121.1(4)	C6	C7	C1	122.7(3)
C2	N1	O2	120.5(3)	C9	C8	N4	129.5(3)
O5	N2	O4	123.3(3)	C13	C8	N4	109.1(3)
C7	N2	O4	117.9(3)	C13	C8	C9	121.3(3)
C7	N2	O5	118.9(3)	C10	C9	C8	117.3(4)
O7	N3	O6	124.3(3)	C11	C10	C9	122.4(4)
C5	N3	O6	117.4(3)	C12	C11	C10	120.8(4)
C5	N3	O7	118.3(3)	C13	C12	C11	118.6(3)
C19	N4	C8	109.2(3)	C12	C13	C8	119.6(3)
C2	C1	O3	120.2(3)	C14	C13	C8	106.1(3)
C7	C1	O3	124.0(3)	C14	C13	C12	134.3(3)
C7	C1	C2	115.8(3)	C15	C14	C13	133.6(3)
C1	C2	N1	120.6(3)	C19	C14	C13	107.0(3)
C3	C2	N1	116.8(3)	C19	C14	C15	119.3(3)
C3	C2	C1	122.6(3)	C16	C15	C14	118.8(3)
C5	C3	C2	118.4(3)	C17	C16	C15	121.1(3)
C3	C5	N3	119.0(3)	C18	C17	C16	121.4(4)
C6	C5	N3	119.2(3)	C19	C18	C17	117.7(3)
C6	C5	C3	121.8(3)	C14	C19	N4	108.5(3)
C7	C6	C5	118.8(3)	C18	C19	N4	129.7(3)
C1	C7	N2	120.5(3)	C18	C19	C14	121.7(3)

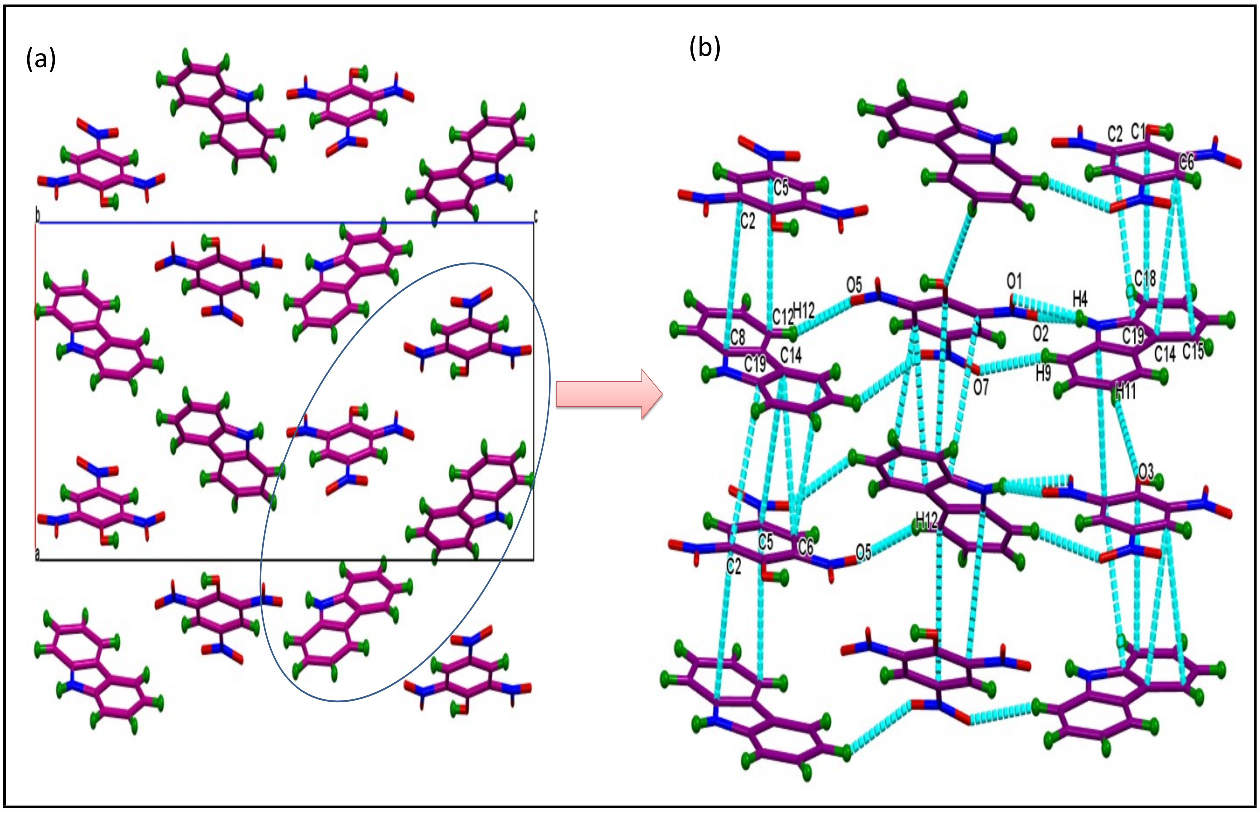

Figure 2: (a) A packing structure of asymmetric unit along with b axis, (b) Asymmetric unit having hydrogen bonding and strong pi-pi interaction form a layered like supramolecular network along the b axis

3.2Topology of CBz-PA

Analysis was performed with the ToposPro program package and the TTD collection of periodic network topologies.[20] The RCSR three-letter codes[21] were used to designate the network topologies. Those nets that are absent in the RCSR are designated with the Topos NDn nomenclature,[22] where N is a sequence of coordination numbers of all non-equivalent nodes of the net, D is the periodicity of the net (D=M, C, L, T for 0-,1-,2-,3-periodic nets), and n is the

ordinal number of the net in the set of all non-isomorphic nets with the given ND sequence. The structure consists of carbazole and picric acid; they form hydrogen-bonded dimers $C_6H_3N_3O_7 \cdot C_{12}H_9N$, do not connect. Hydrogen-bonded fragments form a 0D net, so there are no infinite pathways for proton transport (Figure 3).

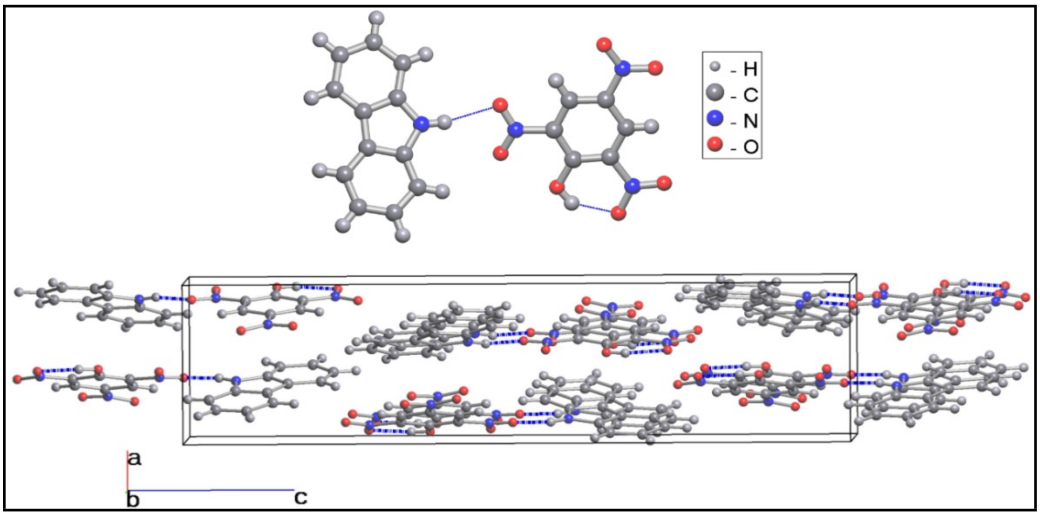

Figure 3: The hydrogen-bonded dimer of carbazole and picric acid (top) and the packing of the dimers in the unit cell (bottom). The hydrogen bonds are highlighted in blue

Standard simplification procedure of the Coulomb or van der Waals bonded structure resulted in the 14,14T584 molecular packing net, with 14-coordinated nodes corresponding to picric acid and carbazole, respectively (Figure 4).

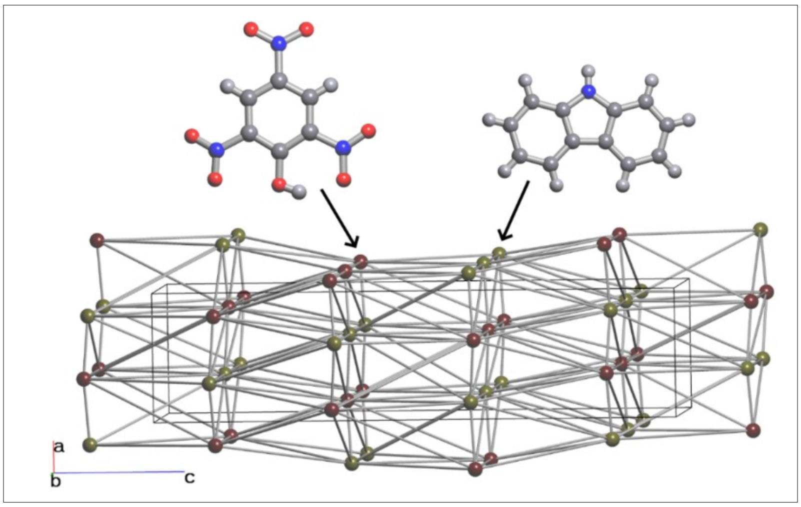

Figure 4: Underlying net of the van der Waals bonded structure. Brown spheres correspond to the picric acid, green carbazole

To conduct the multilevel topological description[23] of the molecular packing, we select the value of molecular solid angle (Ω_i) as a criterion, which will serve as a weight factor proportional to the strength of the intermolecular contact. Using the subroutine Generate Representations implemented in ToposPro, different subnets can be obtained from the underlying net that contains the edges of weight no less than a specified value. The 14-c molecular packing net describes the way molecules are assembled. Each subnet of this net contains information about the spatial arrangement of strong intermolecular contacts, assuming the Ω_i is proportional to the strength of intermolecular interaction (Table 4).

Table 4: Values of the molecular solid angle (Ω_i) and corresponding topological types of the subnets of the molecular packing net

No. of molecules	Ω_i, %	Topology	No. of molecules	Ω_i, %	Topology
14,14	1.49	14,14T584	7,7	5.67	**vcj**
12,14	2.11	12,14T1604	5,7	6.65	5,7T98
12,12	3.87	12T11	5,5	7.18	**nov**
10,12	4.21	10,12T2019	4,4	7.45	**sql**
10,10	4.80	10T11	3,3	9.13	$4^4(0,2)$
9,9	4.98	9,9T240	2,2	9.51	2C1
8,8	5.56	**Ecu**	1,1	16.15	1M2-1

Besides, interactions between carbazole and picric acid play an important role in structure formation. Interaction between molecules appears at the value of Ω_i (16.15%) (Table 4). Thus, we can consider the dimer carbazole and picric acid (Figure 5) as the building unit of the structure.

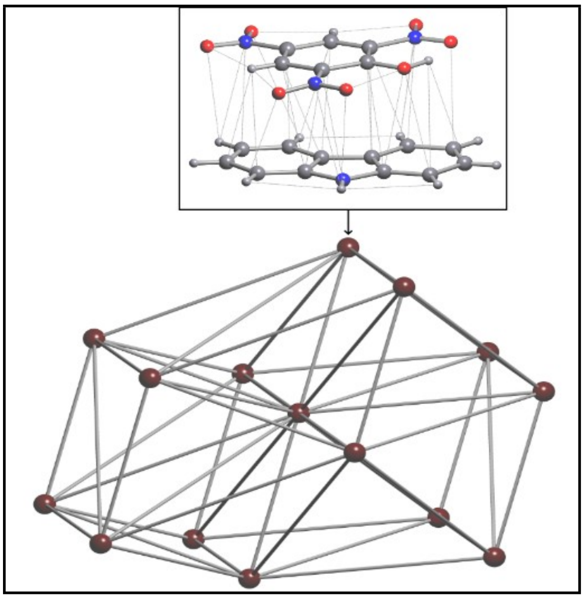

Figure 5: Underlying net of π-bonded dimers packing. Brown spheres correspond to the dimers centroids

The dimers form a 14-coordinated net with a 14T3 topology (Figure 5). Using the subroutine Generate Representations implemented in ToposPro, different subnets can be obtained from the underlying net that contains the edges of weight no less than a specified value. Information about the Ω_i, dimensionality, and topology of corresponding subnets is given in Table 5.

Table 5: Values of the molecular solid angle (Ω_i) and corresponding topological types of the subnets of the π-bonded dimer packing net

**No. of molecules**	**Ω_i, %**	**Topology**
14	3.39	14T3
12	4.12	**Fcu**
10	6.35	**Chb**
8	6.99	**Tsi**
6	7.89	**Lla**
4	9.65	**Sql**
2	11.62	2C1

If we consider the hydrogen-bonded dimer (Figure 5) as a structural building unit, then the topology of the underlying net is 16T3 in the standard representation of the Coulomb or vdW-bonded molecular structures. Information about the Ω_i, dimensionality, and topology of corresponding subnets is given in Table 6.

Table 6: Values of the molecular solid angle (Ω_i) and corresponding topological types of the subnets of the hydrogen-bonded dimer packing net

**No. of molecules**	**Ω_i, %**	**Topology**
16	3.06	16T3
14	3.11	tcg-x
12	3.36	12T31
10	3.72	10T75
8	3.86	8T26
6	4.02	**Bsn**
4	9.57	**Sql**
2	19.3	2C1

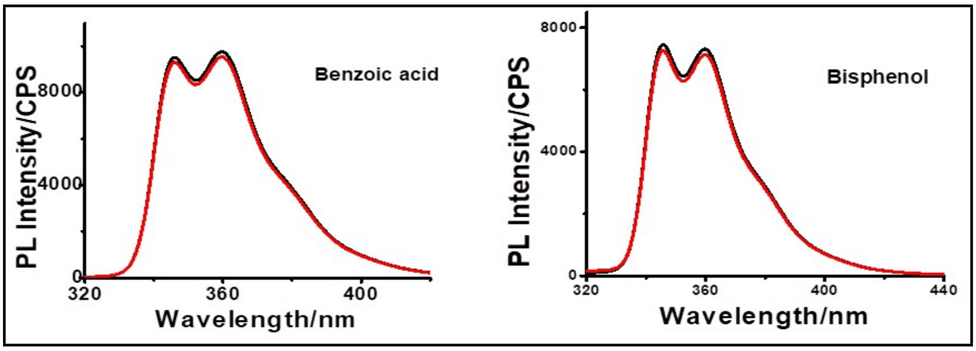

Figure 6: Fluorescence quenching with benzoic acid and bisphenol
